# Evaluation of promoter methylation status of MLH1 gene in Iranian patients with colorectal tumors and adenoma polyps 

**Published:** 2017

**Authors:** Ashkan Zarandi, Shiva Irani, Sanaz Savabkar, Vahid Chaleshi, Maryam Ghavideldarestani, Reza Mirfakhraie, Mahsa Khodadoostan, Ehsan Nazemalhosseini-Mojarad, Hamid Asadzadeh Aghdaei

**Affiliations:** 1 *Department of Biology, Science and Research Branch, Islamic Azad University, Tehran, Iran.*; 2 *Basic and Molecular Epidemiology of Gastrointestinal Disorders Research Center, Research Institute for Gastroenterology and Liver Diseases, Shahid Beheshti University of Medical Sciences, Tehran, Iran.*; 3 *Department of Medical Genetics, Shahid Beheshti University of Medical Sciences, Tehran, Iran.*; 4 *Department of Gastroenterology and Hepatology, Isfahan University of Medical Sciences, Isfahan, Iran.*; 5 *Gastroenterology and Liver Diseases Research Center, Research Institute for Gastroenterology and Liver Diseases, Shahid Beheshti University of Medical Sciences, Tehran, Iran *

**Keywords:** Colorectal polyp, *MLH1*, Promoter methylation, MSP

## Abstract

**Aim:**

The aim of this study was to evaluate the methylation status of the promoter region of MLH1 gene in colorectal cancer (CRC) and its precursor lesions as well as elucidate its association with various clinicopathological characteristics among Iranian population.

**Background:**

Epigenetic silencing of mismatch repair genes, such as* MLH1*, by methylation of CpG islands of their promoter region has been proved to be an important mechanism in colorectal carcinogenesis.

**Methods:**

Fifty colorectal cancer and polyp tissue samples including 13 Primary colorectal tumor and 37 Adenoma polyp samples were enrolled in this study. Methylation-specific polymerase chain reaction (MSP) was performed to find the frequency of MLH1 Promoter Methylation.

**Results:**

Promoter methylation of *MLH1* gene was detected in 5 out of 13 tumor tissues and 4 out of 37 adenoma polyp. The frequency of *MLH1* methylation in tumor samples was significantly higher compared to that in polyp tissues (P= 0.026). No significant association was observed between *MLH1* promoter methylation and clinicopathological characteristics of the patients.

**Conclusion:**

The frequency of *MLH1* promoter methylation in CRC and colon polyp was 18%. Our findings indicated that methylation of *MLH1* promoter region alone cannot be considered as a biomarker for early detection of CRC.

## Introduction

 Colorectal cancer (CRC) is the third most commonly diagnosed malignancy and the fourth leading cause of cancer-related death in the world, accounted for about 1.4 million new cases and 694000 deaths in 2012 (1). In this regard, CRC is the third most common cancer in Iran and it has become a major public health issue due to its increasing incidence in the past three decades (2). 

Similarly to other cancer types, CRC develops as the result of both genetic and epigenetic alterations (3). Chromosomal instability (CIN), CpG island methylator phenotype (CIMP), and microsatellite instability (MSI) are three major pathways of tumourigenesis which have been characterized in CRC (4, 5).

Genome wide instability of short tandemly repeated DNA sequences, referred to as MSI, is observed in 90% of Lynch syndrome patients (6) as well as 10-15% of sporadic cases of colorectal carcinoma (7, 8). Mismatch repair (MMR) is a conserved DNA repair pathway that plays an essential role in maintaining genomic stability (9). Inactivation, mutational and/or epigenetic silencing of MMR genes lead to the MSI phenomenon (10). *MLH1* is one of the key genes in MMR system. Any alterations in the expression of *MLH1* may increase the risk of CRC (11).

Promoter CpG island hypermethylation of certain tumor suppressor genes has been identified to be an important gene silencing mechanism during malignant transformation (12). Multiple studies have indicated that methylation of the *MLHI* promoter region is associated with MSI-positive phenotype (9,13). Moreover, the association between *MLH1* promoter methylation and the risk of CRC has been evaluated in previous studies (14, 15); however, the results are inconsistent. The aim of the present study was to determine the methylation status of *MLH1* gene promoter in colorectal tumor tissues as well as colorectal polyps in Iranian population. 

## Methods

**Sample collection**

Primary colorectal tumor (n=13) and polyp (n=37) samples were collected from Iranian patients who underwent colonoscopy at the Taleghani hospital (Tehran, Iran) during November 2011 to April 2013. Written informed consent was obtained from all participants. The demographic information of all the patients as well as the size, type and location of their polyp or tumor were collected by a physician. The clinical and pathological characteristics of patients are shown in Table 1. None of the patients received chemotherapy or radiotherapy. Moreover, none of the patients had a history of surgery and/or non-colorectal cancers. All the colorectal tissue samples, including polyp, tumor and normal adjacent tissues, were examined by two expert pathologists. All the samples were immediately frozen at -80°C until being used for DNA extraction. This study was conducted under the approval of the ethics committee of the Gastroenterology and Liver Diseases Research Center, Shahid Beheshti University of Medical Sciences, Tehran, Iran with the ethic number of 1392/704.

**Table 1:** Associations between *MLH1* promoter methylation and clinicopathological features of the patients. M* and U** indicate the methylated and un-methylated *MLH1*, respectively

Patient		Total	*MLH1* M*	*MLH1* U**	*P* Value	Fisher's exact test
Gender	Male	24 (%48)	4 (%16.7)	20 (%83.8)	0.814	1.00
	Female	26 (%52)	5 (%19.2)	21 %(80.8)		
Age	≥50	36 (%72)	6 (%16.7)	30 (%83.3)	0.649	0.697
	<50	14 (%28)	3 (%21.4)	11 (%78.6)		
BMI	Normal	19 (%38)	4 (%20)	16 (%80)	0.764	1.00
	Abnormal	31 (%62)	5 (%16.7)	25 (%83.3)		
Pathology status	Adenoma	41 (%82)	8 (%19.5)	33 (%80.5)	0.552	1.00
	Hyperplasia	9 (%18)	1 (%11.1)	8 (%89.9)		
Differentiation	Free of dysplasia	26 (%52)	7 (%26.9)	19 (%73.1)	0.358	NA
	Low-grade dysplasia	9 (%18)	1 (%11.1)	8 (%88.9)		
	Moderate dysplasia	5 (%10)	0 (%0)	5 (%100)		
	High-grade dysplasia	10 (% 20)	1 (%10)	9 (%90)		
Location	Colon	28 (%56)	4 (%14.3)	24 (%85.7)	0.441	0.481
	Rectum	22 (%44)	5 (%22.7)	17 (%77.3)		
Size	<10mm	19 (%38)	1 (%5.3)	18 (%94.7)	0.066	0.127
	≥10mm	31 (%62)	8 (%25.8)	23 (%74.2)		
Types of neoplasm	Polyp	37 (%74)	4 (%10.8)	33 (%89.2)	0.026	0.040
	Tumor	13 (%26)	5 (%38.5)	8 (%61.5)		

**Figure 1:**
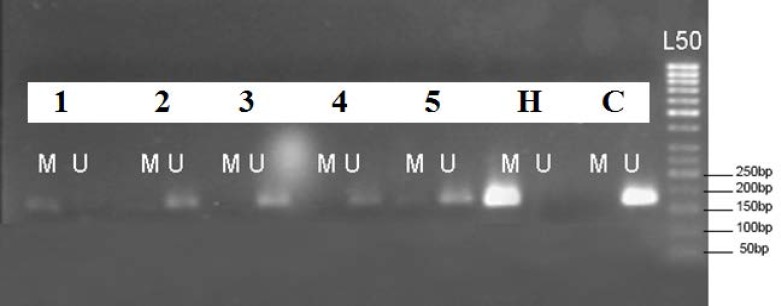
Representative example of *MLH1* promoter methylation analysis in colon neoplasms. The MSP products were loaded and electrophoresed on a 2% agarose gel as follows: Numbers 1-5: DNA products (lanes 1-2: DNA samples extracted from tumor tissues; lanes 3-5: DNA samples extracted from polyp tissues), H: HeLa and C: Control. All MSP products were of the predicted size (170 bps). A 50-bp ladder (L50) served as a reference of measurement. The presence of visible bands wherever marked with U and M, indicated unmethylation and hypermethylation in *MLH1* promoter region, respectively

**DNA extraction and bisulfite modification**

The QIAamp DNA mini kit (Qiagen, Hilden, Germany) was used to extract DNA from the tissues examined in the present study. The extracted DNA was subjected to bisulfite modification using the Qiagen EpiTect Bisulfite kit (Qiagen, Hilden, Germany) according to the manufacturer's instructions. 

**Methylation-specific polymerase chain reaction (MSP)**

The methylation status of *MLH1* gene was investigated using methylation-specific PCR (MSP) technique (16). The MSP primers, specific to the human *MLH1* gene, were selected from the literature (17). PCR was carried out in 12.5 μl reaction mixtures containing 1.5 μl bisulfite-treated template DNA, 1.25 μl 10x MSP PCR buffer, 0.25 μl dNTPs (10 mM), 0.5 μl of each sense and antisense primers (10 μM), 1.25 units GoTaq Flexi DNA polymerase (Promega, Madison, WI, USA) (0.25 μl Taq + 4.75 μl H_2_O), and 14.25 μl H_2_O. The PCR thermal condition was as follows: 15 min denaturation at 95°C, 39 amplification cycles comprising of 1 min denaturation at 95°C, 30 sec annealing at 48.8°C, and 1 min extension at 72°C, followed by an extended elongation for 10 min at 72°C. Details of the primers are listed in Table 1. The PCR products were electrophoresed on 2% agarose gels and evaluated under ultraviolet light.

**Table 2:** The MSP primer sequences for the human *MLH1* gene

Annealing Temperature (°C)	%CG	Length (bp)	Primer sequence	*MLH1* gene
56	34	23	5´-TATATCGTTCGTAGTATTCGTGT-3´	M-Forward
59	50	20	5´-TCCGACCCGAATAAACCCAA-3´	M-Reverse
60	27	29	5´-TTTTGATGTAGATGTTTTATTAGGGTTGT-3´	U-Forward
62	45	24	5´-ACCACCTCATCATAACTACCCACA-3´	U-Reverse

**Statistical analysis**

The statistical analyses of *MLH1* methylation and clinicopathological findings were performed using Pearson’s chi-squared (Χ2) test, Student’s *t-*test and Fisher’s exact test. *P* values computed were two-tailed, and* P*< 0.05 was considered statistically significant. Data were analyzed using SPSS software, version 21 (IBM, Inc., Chicago, Illinois). 

## Results

The present study has evaluated the methylation status of the *MLH1* gene in colorectal tumors and polyps in an Iranian population (Figure 1). Furthermore, the association between the* MLH1* aberrant methylation and demographic as well as clinical characteristics has been investigated in this study (Table 2). No significant correlation was found between *MLH1* methylation and demographic factors, such as gender, age, and BMI. The frequency of *MLH1* promoter methylation was significantly higher in tumor tissues (38.5% or 5/13) compared to that in the polyps (10.8% or 4/37) (P= 0.026). Furthermore, examining the correlation between the *MLH1* hypermethylation and other clinical features of the patients, such as the location and size of the neoplasms, revealed no statistically significant association.

## Discussion

Aberrant DNA methylation of CpG-rich promoter regions of many genes, which results in transcriptional inactivation, has been found to be associated with human colorectal tumorigenesis (18). Therefore, aberrantly methylated genes are promising and valuable biomarkers for prognosis and early detection of CRC (19). *MLH1* gene, as one of the MMR genes, has been proved to be responsible for a substantial portion of colorectal cancer cases with microsatellite instability. Studies have shown that silencing of *MLH1 *by abnormal promoter hypermethylation is the major cause of MSI in sporadic colorectal cancer (20). Therefore, *MLH1* methylation may be a potential biomarker for detection of colorectal neoplasms (17). In view of these observations, we conducted this case-control study to evaluate the promoter hypermethylation status of *MLH1* gene in colorectal cancer and its precursor lesions among an Iranian population and to elucidate its association with various clinicopathological characteristics. In the current study, the methylation status of *MLH1* promoter region, in fresh-frozen tumor and polyp colorectal tissues of 50 patients, was evaluated using MSP technique. The results indicated that *MLH1* gene was hypermethylated in 38.5% (5 out of 13) of tumors and 10.8% (4 out of 37) of polyp samples. In a cohort study conducted by Kim *et al.* (2014) on 33 MSI-H colorectal cancer cases, it has been found that *MLH1* promoter was methylated in 36.4% of patients (21). The higher frequency reported in their study may be due to the fact that *MLH1* promoter methylation is the main cause of MSI-H in sporadic CRC (20). Investigating the association between SNPs and methylation status of the *MLH1* promoter region by Miyakura *et al.* (2014) has revealed a methylation frequency of 28.6% (60 out of 210) in colorectal carcinoma tissues (22). The results of the current study are contradictory to those reported by Sidelnikov *et al.* (2009) who have also investigated the effectiveness of screening CRC patients using *MLH1* methylation as a biomarker. They have suggested that the increased risk of incident, sporadic colorectal adenoma, and other alterable risk factors of colorectal neoplasms may be associated with lower expression levels of *MLH1 *and *MSH2 *in normal colonic mucosa (17). Further analyses of the data obtained in this study have demonstrated that the frequency of *MLH1* promoter methylation was significantly higher in tumors compared to polyp tissues (P= 0.026). One hypothesis, that might explain this difference, would be that methylation of *MLH1* is a late event in colorectal tumorigenesis. Several previous studies have evaluated the relationship between methylation of *MLH1* and clinicopathological features, such as age, gender, tumor location and differentiation (20, 23). However, their findings have been inconsistent. In this regard, Mirchev *et al.* (2007) have reported a significant association between methylation of the *hMLH1* gene and proximal tumor location as well as tumor cells differentiation (23). In line with this, higher frequency of *MLH1* methylation in females, proximal tumor location and low differentiation have been reported in other studies (20). On the contrary, the results of the present study have indicated no significant association between the *MLH1* promoter methylation status and clinicopathological parameters among the patients.

Our findings have revealed that *MLH1* is frequently methylated in cancerous tissues of Iranian CRC patients, but not in the polyps and normal adjacent tissues. Further studies are required to investigate *MLH1* methylation status in a larger number of early and advanced polyp tissues in ordSer to clarify whether *MLH1* methylation has the potential to be used as a diagnostic and prognostic biomarker in colorectal cancer.
